# An Unusual Presentation of Adult-Onset Still’s Disease as Hemophagocytic Lymphohistiocytosis in a Male Patient

**DOI:** 10.7759/cureus.11139

**Published:** 2020-10-24

**Authors:** Neenu Kuruvilla, Rahul Rajendran, Shilpa S Thomas, Irshad Ali KM, Sheela Kurian

**Affiliations:** 1 Department of Internal Medicine, Government Medical College, Kottayam, IND

**Keywords:** adult onset stills disease, hemophagocytic lymphohistiocytosis (hlh), unusual, autoimmune

## Abstract

Hemophagocytic lymphohistiocytosis (HLH) is an aggressive and potentially fatal condition characterized by immune activation and multi-organ dysfunction. HLH can be inherited in an autosomal recessive fashion, but can also be secondary to infections, malignancy, immunosuppression, and autoimmune conditions. Adult-onset Still’s disease (AOSD) is an autoimmune disorder that can predispose patients to HLH. AOSD, similar to other autoimmune conditions, is more common in females than males. However, the occurrence of AOSD in males and subsequent predisposition to HLH is rarely reported. We report the case of a 23-year-old male patient who presented with fever, joint pain, and rash for 20 days. On evaluation, he fulfilled the diagnostic criteria for AOSD and HLH, and a diagnosis of HLH secondary to AOSD was made. He was treated with pulse dose steroids and gradually tapered. AOSD and HLH have overlapping clinical and laboratory features and hence their co-occurrence poses diagnostic challenges. The mortality rate of HLH is high and hence prompt initiation of treatment is of utmost importance.

## Introduction

Hemophagocytic lymphohistiocytosis (HLH) is an aggressive and life-threatening condition of excessive immune activation leading to multi-organ dysfunction. HLH can be primary/familial or secondary to infections, malignancy, immunosuppression, and autoimmune conditions [1]. Common manifestations of HLH include prolonged fever, hepatosplenomegaly, pancytopenia, and elevated levels of liver enzymes, ferritin, and triglycerides [1]. Adult-onset Still’s disease (AOSD) is an autoimmune condition that can predispose patients to HLH. The occurrence of AOSD in males, and subsequent predisposition to HLH, is rare [2]. In this report, we present a rare case of a young male patient with AOSD, who presented with HLH.

## Case presentation

A 23-year-old Indian male, with no significant past medical history and family history, presented to our hospital with fever, joint pain, and rash for 20 days. He had intermittent high-grade fever with chills, which was associated with headache and myalgia. He also reported joint pain involving both elbows, knees, and ankles, with restriction of movement. The patient had an associated evanescent rash involving the trunk. There was no history of vomiting, loose stools, constipation, abdominal pain, chest pain, shortness of breath, palpitations, syncope, seizures, weight loss, or morning stiffness of joints. There was also no history of travel outside of state or exposure to pets. He denied any history of smoking, excessive alcohol use, or substance abuse. On examination, the patient was conscious and oriented with a temperature of 101 °F, pulse rate of 82/min, blood pressure of 120/84 mmHg, and SpO_2_ of 98% on room air. Physical examination showed the presence of non-tender axillary and cervical lymphadenopathy with a salmon-colored maculopapular rash on the skin over the back of the chest. Physical examination was otherwise unremarkable.

Labs at presentation (Table 1) were significant for leukocytosis, erythrocyte sedimentation rate (ESR), and C-reactive protein (CRP).

**Table 1:** Labs at presentation MCV: mean corpuscular volume; ESR: erythrocyte sedimentation rate; CRP: C-reactive protein; AST: aspartate transaminase; ALT: alanine transaminase; ALP: alkaline phosphatase; PT: prothrombin time; INR: international normalized ratio; hpf: high power field; RBC: red blood cell

Variable	Measurement	Reference values
Hemoglobin (g/dL)	13.7	13.5–17.5
Total leucocyte count (TLC) (/mm^3^)	240,000	4,500–11,000
Neutrophil (%)	74	54–62
Lymphocytes (%)	23	25–33
Platelet count (/mm^3^)	381,000	150,000–400,000
MCV (μm^3^)	91	80–100
ESR (mm/h)	110	0–15
CRP (mg/dL)	82.5	≤0.8
Sodium (mEq/L)	132	136–145
Potassium (mEq/L)	4.1	3.5–5.0
Calcium (mEq/L)	8.4	8.6–10.2
Magnesium (mEq/L)	2.3	1.6–2.6
Blood urea nitrogen (mmol/dL)	30	8–24
Creatinine (mg/dL)	1.0	0.6–1.2
Total protein (g/dL)	7.0	6.0–7.8
Albumin (g/dL)	3.6	3.5–5.5
Total bilirubin (mg/dL)	0.6	0.1–1.0
Direct bilirubin (mg/dL)	0.2	0.0–0.3
AST (U/L)	97	8–40
ALT (U/L)	141	8–40
ALP (U/L)	184	30–100
PT (seconds)	17.5	11–15
INR	1.37	0.8–1.2
Urine: albumin, sugar	Nil	
Urine: pus cells	1-2/hpf	
Urine: RBC	Nil	

Infectious disease panel including human immunodeficiency virus (HIV), hepatitis B surface antigen (HBsAg), hepatitis C virus (HCV), syphilis, Widal test, immunoglobulin M (IgM) scrub typhus, brucellosis, leptospirosis, and dengue fever were negative. Peripheral smear was normal except for a few transformed lymphocytes. Direct and indirect Coombs test and blood and urine culture also came back normal. Ultrasound of abdomen and pelvis showed hepatosplenomegaly. The initial differential diagnoses were inflammatory or autoimmune conditions and malignancy. The patient was started on pulse dose steroids with intravenous methylprednisolone 1,000 mg daily after sending the right axillary lymph node for histopathological examination. Biopsy of the lymph node showed atypical mononuclear cells and histiocytes with hemophagocytosis. His antinuclear antibody (ANA) profile, ferritin, and lactate dehydrogenase (LDH) were evaluated, which revealed elevated levels of ferritin and LDH (Table 2). The patient met the diagnostic criteria for AOSD and HLH, and a diagnosis of HLH secondary to AOSD was made.

**Table 2:** Autoimmune and HLH panel LDH: lactate dehydrogenase; ANA IF: anti-nuclear antibody immunofluorescence; HLH: hemophagocytic lymphohistiocytosis

Variable	Measurement	Reference values
LDH	1,240	45–90 U/L (100–250 IU/L)
Ferritin (ng/mL)	1,532	15–200
ANA IF	Negative	
Rheumatoid factor	Negative	
S. Fibrinogen	355	200–400
Total cholesterol (mg/dL)	246	<200
Fasting triglycerides (mg/dL)	280	<150

His general condition improved over the next week, and so he was switched to oral prednisolone 60 mg once daily. There were no more episodes of fever, and he was discharged after one week on a tapering dose of steroids.

## Discussion

We reported a case of a young male patient who presented with HLH secondary to AOSD. His clinical and laboratory findings included fever, arthralgia, salmon-colored rash, lymphadenopathy, hepatosplenomegaly, leukocytosis, abnormal liver function test (LFT), and negative ANA and rheumatoid factor (RF), thereby meeting eight out of the nine Yamaguchi criteria for the diagnosis of AOSD [3]. He also had hemophagocytosis (detected via lymph node biopsy) and hyperferritinemia, fulfilling five out of the eight diagnostic criteria of HLH as described in the HLH-2004 trial [4,5]. Hence the patient was diagnosed with HLH complicated by AOSD.

AOSD is an autoimmune condition similar to the one observed in children (systemic-onset juvenile idiopathic arthritis). AOSD has a bimodal age distribution: 15-25 and 36-46 years of age [6]. The exact etiology of AOSD is still not clear. Several factors such as infections (bacterial, viral), genetic factors, and immune dysfunctions have been suggested [6]. Pathologically, AOSD involves the excess activity of interleukin 1 (IL-1), IL-6, IL-8 interferon-gamma (IFNγ), tumor necrosis factor-alpha (TNFα), C-X-C motif chemokine ligand 10 (CXCL-10), and CXCL-13 [7-9]. AOSD is characterized by high-grade quotidian fever (≥39°C), non-suppurative pharyngitis, transient rash (nonpruritic, salmon-colored, and maculopapular, and often observed during febrile episodes) involving the trunk and proximal extremities, and arthralgia, commonly involving the knees, wrists, ankles, and elbows [6]. Laboratory findings in AOSD are similar to those in other autoimmune conditions [elevated white blood cell (WBC) count, abnormal LFTs, and elevated levels of CRP, ESR, and ferritin]. RF and ANA are usually negative in AOSD. AOSD is primarily a diagnosis of exclusion, which is arrived at after excluding infections (viral and bacterial), malignancies, vasculitis, and other connective tissue diseases [6]. HLH is one of the most serious complications of AOSD, and both of them share clinical and laboratory features and hence pose diagnostic challenges [10]. Unlike AOSD, HLH may have leukopenia/thrombocytopenia, and very high levels of serum triglycerides. Also, the levels of fibrinogen may be normal or low in AOSD patients who develop HLH, as seen in our patient [11].

HLH can be primary/familial resulting from a genetic defect leading to the decreased cytotoxic activity of natural killer (NK) cells and cytotoxic T cells [12]. Secondary HLH is precipitated by infections, autoimmune conditions, or malignancies. HLH involves inappropriate activation of T cells and macrophages, which produces pro-inflammatory cytokines, progressing to cytokine storm and resulting in organ dysfunction [1,13]. The treatment of HLH is based on the HLH-94 protocol [14]. Induction therapy consists of etoposide (twice weekly during the first two weeks, and then weekly), in combination with dexamethasone. Central nervous system (CNS) disease is treated with intrathecal methotrexate. The HLH-2004 protocol was initiated in 2004 [5]. It differs from the HLH-94 protocol in that it recommends using cyclosporine from day one of the initial treatment phases, and involves the addition of intrathecal steroids to intrathecal methotrexate for CNS disease [5]. Treatment of secondary HLH is aimed at treating the underlying condition.

The treatment of AOSD involves anti-inflammatory drugs including non-steroidal anti-inflammatory drugs (NSAIDs), steroids, and anti-rheumatic agents including methotrexate, azathioprine, cyclosporine, and cyclophosphamide for symptom control [15]. Anti-interleukin 1 (IL-1Ra and anakinra), anti-interleukin 6 (tocilizumab), TNF-α inhibitors (infliximab, etanercept, and adalimumab), and anti-IL1β (canakinumab) are the biological agents shown to be effective in the treatment of AOSD [6].

AOSD has a variable prognosis. It can be self-limited, characterized by fever and rash, and remission usually occurs within one year of the disease onset. The patients can also experience intermittent flare-up episodes with or without joint involvement. The disease can also have a chronic course with joint involvement, finally resulting in joint destruction [6].

## Conclusions

AOSD in males and AOSD predisposing patients to HLH are rare. Their co-occurrence is associated with overlapping clinical and laboratory features and hence a high level of suspicion is necessary for diagnosis. A high index of suspicion is important for the diagnosis of HLH and prompt initiation of treatment is of utmost importance, as it is a rapidly progressive life-threatening condition.
